# Combined Water Extracts from Oxidation-Treated Leaves and Branches of *Hovenia dulcis* Has Anti-Hangover and Liver Protective Effects in Binge Alcohol Intake of Male Mice

**DOI:** 10.3390/nu13124404

**Published:** 2021-12-09

**Authors:** Jihyun Je, Miyoung Song, Ji Hyeong Baek, Jae Soon Kang, Hye Jin Chung, Kwonsu Lee, Sang Won Park, Hyun Joon Kim

**Affiliations:** 1 Department of Pharmacology, Institute of Health Sciences, Anti-Aging Bio Cell Factory Regional Leading Research Center, Gyeongsang National University Medical School, 15 Jinju-daero 816 Beongil, Jinju 52727, Gyeongnam, Korea; jeri1984@naver.com; 2 Department of Anatomy and Convergence Medical Sciences, Institute of Health Sciences, Anti-Aging Bio Cell Factory Regional Leading Research Center, Gyeongsang National University Medical School, 15 Jinju-daero 816 Beongil, Jinju 52727, Gyeongnam, Korea; mysong0626@naver.com (M.S.); baekbaek@gnu.ac.kr (J.H.B.); jskang@gnu.ac.kr (J.S.K.); 3 College of Pharmacy and Research Institute of Pharmaceutical Sciences, Gyeongsang National University, 501 Jinju-daero, Jinju 52828, Gyeongnam, Korea; hchung@gnu.ac.kr; 4 Malgeunsan Agricultural Union Corp., 991 Wolasan-ro, Munsan-eup, Jinju 52839, Gyeongnam, Korea; malgeunsan@naver.com

**Keywords:** alcohol-induced liver injury, flavonoid catechin, anti-hangover, oxidative/nitrosative, *Hovenia dulcis*

## Abstract

*Hovenia dulcis*, known as the oriental raisin tree, is used for food supplements and traditional medicine for the liver after alcohol-related symptoms. However, little information exists about the use of its leaves and branches. In this study, we established a method to use the leaves and branches to develop anti-hangover treatment and elucidated the underlying mechanisms. Oxidation-treated leaves (OL) exhibited high antioxidant content comparable to that of the peduncles and showed an anti-hangover effect in male mice. The branch extract (BE) was enriched in the flavonoid catechin, approximately five times more than OL extract. The mixture of OL and BE (OLB) was formulated in a 2:1 ratio with frozen-dried extract weight and was tested for anti-hangover effects and protective properties against binge alcohol-induced liver injury. OLB showed better anti-hangover effect than OL. In addition to this anti-hangover effect, OLB protected the liver from oxidative/nitrosative damage induced by binge alcohol intake.

## 1. Introduction

Alcoholic beverages have been enjoyed by many people for thousands of years all over the world. Alcohol-induced liver injury is one of the most common causes of liver diseases worldwide. Therefore, some natural products with antioxidant activity have attracted great attention as potential functional ingredients to protect alcohol-induced liver injury [1,2,3]. Extracts from *Hovenia dulcis*, the oriental raisin tree, have been used widely to relieve alcohol toxicity as substances for functional food and beverages before and after alcohol drinking from ancient to current times in East Asia [4,5]. Recently, the tree has been widely cultivated for commercial usage, but only the peduncles have been used and traded in the market. Besides the peduncles, almost all leaves and branches are discarded. From the viewpoint of economics, it could be an attractive option to develop a way to use the leaves and branches because they are very inexpensive.

There is no consensus on the definition of an alcohol hangover. However, it is generally recognized as a collection of unpleasant symptoms occurring after the consumption and full metabolism of alcohol. Although a hangover might be considered trivial, it has substantial economic consequences due to decreased productivity and increased risk for injury in the workplace [6]. Frequent hangovers are also associated with increased cardiovascular and psychomotor morbidity [7]. The high prevalence of alcohol hangover is becoming a more important issue: more than 75% of people who consume alcohol have reported that they have experienced a hangover at least once. Interestingly, hangovers are substantially more common in light-to-moderate drinkers than in heavier drinkers [6].

Although alcohol consumption leading to hangover is not a good practice for everyone, binge alcohol drinking is worse, which has toxic effects on liver function of detoxification of hazardous materials, including ingested alcohol and its metabolites [8]. One of these metabolites is acetaldehyde. Alcohol is initially metabolized by alcohol dehydrogenase (ADH) to acetaldehyde, which is rapidly metabolized to acetate by aldehyde dehydrogenase (ALDH). Acetate is a precursor of acetyl CoA, which can be converted to carbon dioxide and water in the Krebs cycle. Hepatic alcohol metabolism plays an important role in alcohol metabolism [9,10]. A previous study reported that a binge alcohol dose (1–5 g/kg body weight) induces alterations in various parameters related with steatosis and necrosis of the liver and suggested that modest liver injury appeared 4 h after acute alcohol treatment [11].

A few trials have been performed to investigate the effects of foods and their constituents on alcohol hangover including red ginseng [12], sprout ginseng [3], artichoke [13], *Opuntia ficus-indica* [14] and *H. dulcis* [5,15]. In the case of *H. dulcis*, there have been a few reports that show preventive and therapeutic properties against alcoholic liver injury but used only the fruits or peduncles [5,15]. However, there is little information about the usage of the tree’s leaves and branches in anti-hangover and anti-liver injury induced by alcohol. There are only trades of raw and extracts of peduncle as functional substances in the current traditional market or industrial food market [16], but there is no evidence for trade in the market and use of leaves and branches. Only minimal information is available in the social network service about their possible functions. Therefore, in the present study, we aimed to develop a way to use the discarded leaves and branches of *H. dulcis* using a newly patented method [17] to exacerbate their antioxidant contents *via* oxidation. The combination of oxidation-treated leaf extract and branch extract showed better suppressive effects for alcoholic hangover and acute liver injury.

## 2. Materials and Methods

### 2.1. Materials

Reference standards ([+]-catechin, chlorogenic acid, ampelopsin, taxifolin, ferulic acid, myricetin, and quercetin) were purchased from Sigma-Aldrich (St. Louis, MO, USA). High-performance liquid chromatography (HPLC) grade water, acetic acid, methanol, and ethanol were from Fisher Scientific (Waltham, MA, USA).

### 2.2. Preparations of Extracts and Characterization

Malgeunsan Agricultural Union Corp. cultivated *H. dulcis* at Sanchung in the Gyeonnam province. The leaves and branches were harvested from October to November in 2021, and leaves were treated using our patented oxidation treatment protocol [18]. Raw leaves (RL) and oxidation-treated leaves (OL; 4 g) were added to water (100 mL) and heated at 95 °C for 3 h for water extracts. The air-dried branches (4 g) were also added to 100 mL water and heated at 95 °C for 3 h. After 4 h heating, hot water extracts were filtered with Whatman # 4 filter paper and freeze dried.

### 2.3. Determination of Total Phenolic and Flavonoid Contents

The total phenolic and flavonoid contents of the extracts were measured as previously described [3] using Folin–Ciocalteu reagent and aluminum chloride, respectively. The total phenolic and flavonoid contents of the samples were expressed as gallic acid equivalent and rutin equivalent, respectively. To determine the contents of five phenolic compounds in the extracts, freeze-dried samples were used. The sample solution for HPLC analysis was prepared by dissolving the freeze-dried extract in ethanol at a concentration of 100 mg/mL and filtering through a 0.2-μm syringe filter. Standard stock solutions of (+)-catechin, chlorogenic acid, taxifolin, ferulic acid, and quercetin were made at a concentration of 1 mg/mL in ethanol. Calibration curves were obtained using calibration standard mixture solutions having concentrations of 0.5, 1, 2, 5, 10, 20, and 50 μg/mL for each component. The sample solutions and calibration standard solutions were analyzed by HPLC.

### 2.4. Animals and Treatments

Male ICR mice (23–25 g) were purchased from KOATECH Co. (Pyeongtaek, South Korea) and maintained in the animal facility at Gyeongsang National University. All animal experiments were approved by the Gyeongsang National University Institution Animal Care & Use Committee (GNU-200820-M0052) and were performed in accordance with the National Institutes of Health guidelines for laboratory animal care. Mice were housed with an alternating 12-h light/dark cycle and were provided with water and standard chow. Mice were fasted for 18 h before experiments. ICR mice were randomly divided into each group. To test the oxidation-treated leaf extract, three groups were employed: vehicle (water), RL, and OL. For combined formula extract of OL and branches, three groups were also employed: vehicle, OL, and OL + branches (OLB). Each freeze-dried extract (0.2 g/kg), the human equivalent dose of 1 g/60 kg bodyweight/day [19], was administered orally 1 h before the intraperitoneal injection of ethanol (4.5 g/kg or 1.5 g/kg). The mice were sacrificed after 1 or 3 h for biochemical analyses.

### 2.5. Righting Reflex Test

Righting reflex test was performed as previously described [3]. In brief, loss of the righting reflex was measured for 80 min after the injection of 4.5 g/kg ethanol. Mice (*n* = 7) were placed on their back following the injection (4.5 g/kg ethanol), and the behavioral changes were scored every 5 or 10 min: normal (score 0), drag one’s hind legs (score 1), return to normal position, but not able to move forward (score 2), and left abnormal position (score 3). Scores were plotted over time, and the area under curve (AUC) was calculated.

### 2.6. Biochemical Assays

Total antioxidant levels were estimated by total antioxidant capacity assay Kit T-AOC (ab65329). Plasma ethanol and acetaldehyde concentrations were measured using kits (ab65343 and ab12113) from Abcam (Cambridge, MA, USA). ADH and ALDH activities were measured using kits (ab102533 and ab155893) from Abcam. Plasma alanine aminotransferase (ALT) and aspartate aminotransferase (AST) activities were measured using kits from IVD Lab (Uiwang, Republic of Korea). All assays were performed according to the manufacturers’ protocols.

### 2.7. Measurement of the GSH/GSSG Ratio and Catalase Activity in the Liver

The glutathione (GSH) to oxidized glutathione (GSSG) ratio and catalase activity of liver tissue were measured using kits (ab205811 and ab83464) from Abcam (Cambridge, MA, USA). Briefly, liver tissues were homogenized in ice-cold phosphate-buffered saline. Then, the homogenized tissues were centrifuged at 4 °C for 15 min. The supernatants were collected and used for measuring the GSH to GSSG ratio and catalase activity using detection kits (ab205811 and ab83464) according to the manufacturer’s protocols.

### 2.8. Immunohistochemistry

Immunohistochemical staining was performed as previously described [3]. In brief, paraffin liver sections (5 µm) were deparaffinized, rehydrated, and antigen retrieved in sodium citrate buffer (10 mM, pH 6.0; iNtRON Biotechnology, Seongnam, Korea) for 20 min. The sections were blocked in 10% normal horse serum (Vector Laboratories, Burlingame, CA, USA) and incubated with a primary antibody for nitrotyrosine (ab61392, Abcam, Cambridge, UK) overnight at 4 °C. Sections were washed and incubated with a biotinylated secondary antibody (Vector Laboratories) for 1 h at room temperature. Sections were washed again and incubated in avidin–biotin–peroxidase complex solution (ABC solution; Vector Laboratories) and then developed using a 3,3′-diaminobenzidine peroxidase substrate kit (Vector Laboratories). The sections were counterstained with hematoxylin and analyzed using a CKX41 light microscope (Olympus, Tokyo, Japan).

### 2.9. RT-PCR Analysis

Total RNA was extracted using Trizol reagent (Thermo Fisher Scientific, Waltham, MA, USA) and converted into cDNA with the RevertAid Reverse Transcription System (Thermo Fisher Scientific) according to the manufacturer’s protocol. Quantitative PCR was performed on the CFX Connect Real-Time PCR System using iQ SYBR Green Supermix (Bio-Rad, Hercules, CA, USA). Relative mRNA levels were normalized to those of *glyceraldehyde 3-phosphate dehydrogenase* (*GAPDH*). The genes and primers for RT-PCR analysis are listed in Table 1.

### 2.10. Statistics

All data are represented as the means ± standard error of the mean (SEM) and statistically analyzed by the one-way analysis of variance (ANOVA) with Tukey’s post hoc test using Prism 5 (GraphPad Software, La Jolla, CA, USA). A *p*-value less than 0.05 was considered statistically significant.

## 3. Results

### 3.1. Water Extract of Oxidation-Treated Leaves from H. dulcis Contains More Antioxidants Than Nontreated Raw Leaves and Has an Anti-Hangover Effect

**Figure 1:**
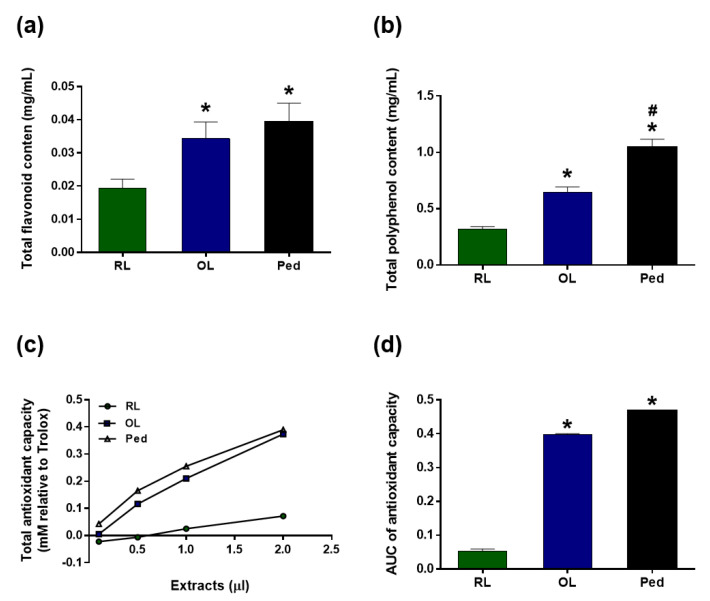
Antioxidant content of raw leaves (RL), oxidation-treated leaves (OL), and peduncles (Ped). Total flavonoid content (**a**) and polyphenol content (**b**). Total antioxidant capacity was measured via the ABTS assay (**c**), and the AUC is represented (**d**). Data are presented as the means ± SEM. * p < 0.05 versus RL; *# p* < 0.05 versus OL.

*H. dulcis* leaves were harvested from October to November from organically grown trees without pesticides in Sancheong, Gyeongnam, Korea. Leaves were washed with freshwater and processed at 40–45 °C for 16–24 h for oxidation treatment [18]. After the process, OL were dried at room temperature and subjected to hot water extraction at 95 °C for 4 h at a concentration of 4% by weight/volume. The water extract was refrigerated at 4 °C until use. RL and peduncles (Ped) were also collected from the same tree and dried to obtain a hot water extract similar to that of the OL. As a result of examining the total flavonoid and polyphenolic compound contents of RL, OL, and Ped, it was confirmed that the amount of both types of compounds were significantly higher in the OL than in the RL (Figure 1a,b). In the case of flavonoids, OL had a similar amount to that from Ped of the same weight (Figure 1a). The ABTS assay was applied to test for the antioxidative activity of those extracts. Expectedly, OL showed the significant higher antioxidative effect than RL, which was comparable to that of Ped.

**Figure 2:**
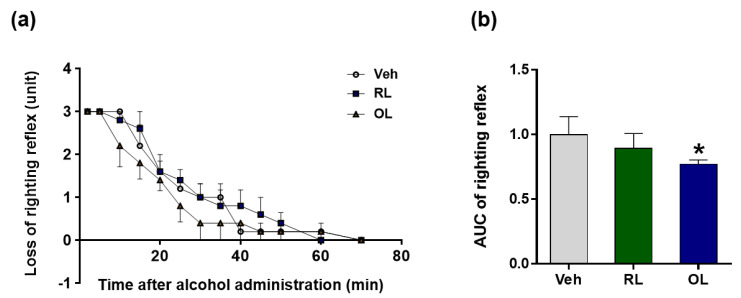
Effect of two extracts, raw leaves (RL) and oxidation-treated leaves (OL), on alcohol tolerance time following acute ethanol-loading in mice. Mice were treated with water (Veh), RL, or OL 1 h before injection of 4.5 g/kg ethanol (*n* = 6, respectively). Loss of stereotactic reflexes was monitored for 80 min, scores of behavioral changes were plotted over time (**a**), and the area under the curve (AUC) was calculated (**b**). Data are presented as the means ± SEM. * *p* < 0.05 versus control.

The anti-alcoholic hangover effects of RL and OL were tested in comparison with the water vehicle control (Veh). Each extract and Veh were administered (10 mL/kg) through gavage 30 min before alcohol (30%) injection; ethanol was injected intraperitoneally (4.5 g/kg). The degree of hangover behavior was measured as previous reported [3]. As a result, it was revealed that RL did not show an anti-hangover effect compared with Veh, consistent with previous reports [16]. However, OL showed a significantly better anti-hangover effect compared with Veh and RL, which is likely due to the higher antioxidative content in the OL than in the RL (Figure 2).

### 3.2. Water Extract of H. dulcis Branches Contains Enriched Catechin Flavonoids

Although it was confirmed that OL had an anti-hangover effect compared with RL, the amount of branches produced during the year is quite large due to the fast-growing trait of *H. dulcis*. Additionally, branches that are less than a year old are softer and have been used for food since ancient times. To develop abandoned forest product, we used the branches and made an extract with hot water. The antioxidant content of the combined OL and branches extract (BE) was examined in freeze-dried powder using HPLC. We examined five polyphenol compounds: (+)-catechin, taxifolin, quercetin, chlorogenic acid, and ferulic acid. HPLC results showed that OL contained all five polyphenols; the concentration of these polyphenols reached as high as approximately 180 µg/g. We could not detect quercetin and chlorogenic acid in branches, despite them being abundant in OL. Interestingly, we confirmed that BE has approximately five times higher (+)-catechin levels than OL (Table 2).

### 3.3. Mixture of Oxidation-Treated Leaves and Branches Extract Shows an Anti-Hangover Effect

Catechin is a representative natural material being enriched in green tea extract and has been known to have antioxidative, antitumor, and anti-alcohol benefits [20,21,22]. We wanted to extend the usage of wasted forest byproducts and develop a way to make functional food with them. To this purpose, we mixed OL and BE in a 2:1 ratio of its weight of freeze-dried powder and completed a comparable experiment with OL and the combined extract of OL and BE (OLB).

**Figure 3:**
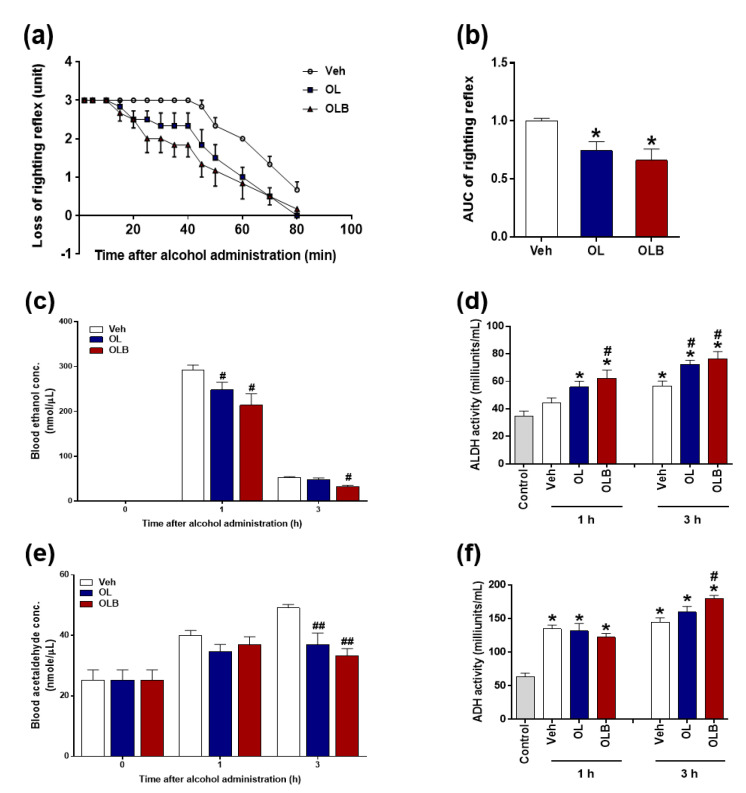
Effect of two extracts, oxidation-treated leaves (OL) and oxidation-treated leaves and branches (OLB), on alcohol tolerance time following acute ethanol-loading in mice. Mice were treated with water (Veh), OL, or OLB 1 h before injection of 4.5 g/kg ethanol (*n* = 6, respectively). Loss of stereotactic reflexes was monitored for 80 min, scores of behavioral changes were plotted over time (**a**), and the area under the curve (AUC) was calculated (**b**). Blood ethanol (**c**) and acetaldehyde (**e**) levels were measured. OL and OLB decreased blood ethanol and acetaldehyde concentrations in ethanol-injected rats. Mice were treated with water, OL, or OLB 1 h before 1.5 g/kg (**c**–**f**) ethanol injection (*n* = 8, respectively), and blood was collected 1 and 3 h later. As a control group, an untreated treatment group (*n* = 5) was included. In ethanol-injected mice, OLB increased the activity of ethanol-metabolizing enzymes. Hepatic activity of alcohol dehydrogenase (ADH) and aldehyde dehydrogenase (ALDH) in liver tissue was measured 1 and 3 h after ethanol injection (**d**,**f**). Data are presented as the means ± SEM. * p < 0.05 versus control, *# p* < 0.05 versus Veh; *## p* < 0.01 versus Veh.

As described in Figure 3, the anti-hangover experiment was conducted with two extracts, OL and OLB. Both extracts showed anti-hangover effects compared with Veh, and there was no difference between the two extracts (Figure 3a,b). Blood alcohol concentration of both extract groups was lower than that of the Veh group after 1 h, and it was significantly lower in the OLB group than in the OL and Veh at 3 h (Figure 3c). For the blood concentration of acetaldehyde, a significant lowering effect was observed in both extracts compared with the Veh group at 3 h (Figure 3e). The activity of alcohol dehydrogenase (ADH) was increased in all groups by alcohol administration, and in particular, the OLB group had more increased activity than the other groups at 3 h. The activity of acetaldehyde dehydrogenase was increased at 1 h in both extracts, and at 3 h, a statistical difference was found in both extract groups compared with the Veh group.

**Figure 4:**
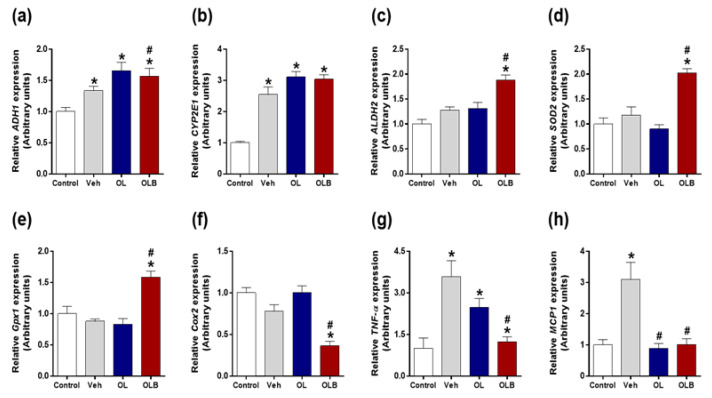
Quantitative real-time PCR analysis of ethanol metabolism–related and oxidative stress genes in the liver tissue of acute ethanol-induced mice. The mRNA levels of *ADH1* (**a**), *CYP2E1* (**b**), *ALDH2* (**c**), *SOD2* (**d**), *Gpx1* (**e**), *Cox2* (**f**), *TNF-α* (**g**), and *MCP1* (**h**) in the liver are shown at 3 h after ethanol injection. Expression level was normalized to that of *glyceraldehyde 3-phosphate dehydrogenase*. Abbreviations: *ADH1*, *alcohol dehydrogenase 1*; *cytochrome p450 enzyme 2E1*, *CYP2E1*; *ALDH2*, *aldehyde dehydrogenase 2*; *SOD2*, *superoxide dismutase 2*; *Gpx1*, *glutathione peroxidase 1*; *Cox2*, *cyclooxygenase 2; TNF-α,* Tumor necrosis factor-α; *MCP1,* *Monocyte chemoattractant protein 1*. Values represent the means ± SEM (*n* = 5 mice for each group). * p < 0.05 versus control, *# p* < 0.05 versus Veh.

The gene expression of *ADH1* and *cytochrome P450 2E1* (*CYP2E1*), representative enzymes converting alcohol to acetaldehyde, were investigated. The mean increment of both genes increased compared to Veh group, and a statistically significant increase in *ADH1* gene expression was observed in the group administered OLB at 3 h compared with the Veh group (Figure 4a,b). The expression of *ALDH2* gene for acetaldehyde degrading enzyme was significantly increased only in the OLB group (Figure 4c).

### 3.4. Combined Formulation of Oxidation-Treated Leaves and Branches Extract Prevents Liver from Binge Alcohol-Induced Liver Injury

**Figure 5:**
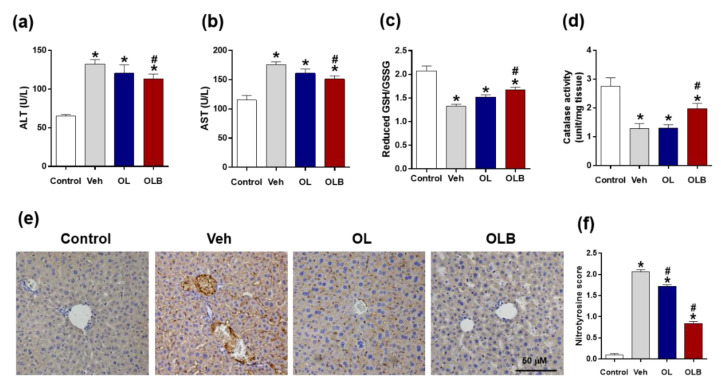
OLB treatment prevents acute ethanol-induced liver damage. Mice were treated with water (Veh), OL, or OLB 1 h before injection of 1.5 g/kg ethanol, and blood and liver tissues were collected 3 h later. As a control group, an untreated treatment group (*n* = 5) was included. Blood was collected, and plasma (**a**) ALT and (**b**) AST levels were measured. The ratio of reduced glutathione (GSH) to oxidized glutathione (GSSG) (**c**) and catalase activity (**d**) in mice liver tissue were measured after ethanol injection. Liver sections from each group were processed for representative immunohistochemistry of nitrotyrosine-positive signals, and representative images are shown (**e**). Nitrotyrosine score was measured (**f**). The data are presented as the means ± SEM. * p < 0.05 versus control; *# p* < 0.05 versus Veh. Scale bar = 50 µm.

The gene expression changes of *superoxide dismutase 2* (*SOD2*) and *glutathione peroxidase 1* (*Gpx1*) related to acute alcohol liver injury were investigated at 3 h after alcohol injection. Interestingly, significant increments were found in the OLB group for *SOD2* and *Gpx1* (Figure 4d,e). We also measured the expression changes of inflammatory related genes, *Cox2*, *TNF-α,* and *MCP 1* [23], and found OLB remarkably suppressed those gene expressions (Figure 4f–h). Alanine transaminase (ALT) and aspartate aminotransferase (AST), landmarks for liver function, were also examined, and OLB administration was found to significantly suppress their activity compared with that of the Veh group at 3 h (Figure 5a,b).

### 3.5. OLB Extract Protects the Liver from Oxidative/Nitrosative Damages Induced by Alcohol

GSH plays an important role in the detoxification of alcohol, and acute alcohol administration leads to GSH depletion in the liver [24,25,26]. Thus, GSH and GSSH levels were measured in mice liver tissue at 3 h after alcohol injection. Expectedly, alcohol reduced the GSH to GSSH ratio, but OLB significantly inhibited the reduction (Figure 5c). ADH, CYP2E1, and catalase all contribute to oxidative metabolism of ethanol [27,28]. Catalase is located in the peroxisome and converts alcohol to acetaldehyde accompany with degradation of hydrogen peroxide into water [29,30]. Although catalase’s role in the metabolism of alcohol is a minor pathway of alcohol oxidation in the normal eating condition, its role is very important in the fasting state [31,32,33]. Alcohol reduced catalase activity, but OLB significantly protected it (Figure 5d). Alcohol metabolism could result in increasing oxidative/nitrosative stress in liver tissue, which increases nitration of tyrosine residues of proteins. Tyrosine nitration changes protein structure and decreases their activity (e.g., catalase) [34,35]. Thus, with anti-nitrotyrosine antibody, immunohistochemical analysis was conducted. We confirmed the increment of nitrotyrosine in liver tissue by alcohol (Figure 5e,f). However, OLB reduced this increment, indicating anti-nitrosative properties, which might be closely related to catalase activity in the OLB group (Figure 5d).

## 4. Discussion

*H. dulcis*, known as the oriental raisin tree, is primarily found in East Asia. It has traditionally been used as an anti-hangover herbal medicine [36]. However, so far, no attention has been paid to the use of its leaves and branches. A previous study reported that peduncle extract yields the highest activity for decreasing blood alcohol concentration among peduncle, leaf, and branch extracts. In addition, peduncle extract significantly inhibited the elevation of serum alanine aminotransferase, aspartate aminotransferase, and lactate dehydrogenase levels [16]. However, this study reported that extract from the leaves and branches did not show any significant activity for decreasing alcohol concentration. Other studies also found the hepatoprotective effects of the peduncle of *H. dulcis* on alcohol-induced liver injury mice via antioxidant activity [37] and suppression of lipopolysaccharide-stimulated inflammatory responses in RAW 264.7 cells [38]. Interestingly, we developed a method to use the leaves and branches of *H. dulcis* via a new established oxidation treatment [18], increasing the antioxidant contents compared with RL (Figure 1a,b). The extract of OL showed remarkable antioxidative performance and anti-hangover activity (Figure 1 and Figure 2), and OLB also showed additional hepatoprotective effect against binge ethanol drinking (Figure 4 and Figure 5). To the best of our knowledge, it is the first report about the usage of leaves and branches of *H. dulcis* for anti-hangover activity and hepatoprotection.

In the present study, serum levels of alanine aminotransferase and aspartate aminotransferase were significantly increased 3 h after alcohol administration (Figure 5a,b). At that time, blood alcohol level decreased as low as 100 nmol/uL, indicating administered alcohol was almost converted into acetaldehyde. The fastest rate of alcohol conversion into acetaldehyde was observed in the OLB group, which is closely related to the increment of ADH activity (Figure 3f). Additionally, blood acetaldehyde levels were increased and accompanied with diminished blood ethanol levels after alcohol administration, but OL and OLB showed significant decrement compared with the vehicle group. This finding would be partially due to the increment of ALDH activity at 1 and 3 h (Figure 3e,d). Although there was no statistical significance, OLB showed better overall performance in anti-hangover activity, blood change of ethanol and acetaldehyde clearance, and activities of ADH and ALDH.

The major differences between the OL and OLB extracts was the amount of catechin flavanoids, which were abundant in the BE (Table 2). This result was very interesting because catechin is the primary flavonoid in green tea, made of leaves of the tea tree, *Camellia sinensis*. However, in *H. dulcis*, catechin is more abundant in the branches than in the leaves. Catechin has recently attracted attention for its antitumor and anti-arteriosclerotic effects [39,40,41,42]. In addition, results of animal experiments have indicated that catechins affect lipid metabolism by decreasing triglyceride and total cholesterol levels [43,44] and enhancing energy utilization [45]. In the present study, OLB containing catechin-enriched BE showed a few additive benefits to the effect of OL. First, OLB increased the expression of *ALDH2* (Figure 4c) and was accompanied by increments of ALDH activity (Figure 3f), contributing to the anti-hangover effect on binge alcohol intake. Second, changes in *SOD2*, *Gpx1*, and *Cox2* found in the OLB group (Figure 4d–f) suggest that OLB intake would have a repertoire to battle against oxidative stress and inflammatory events in the mouse liver. Eventually, the depletion of GSH and activity of ALT/AST were inhibited (Figure 5a–c), indicating the less harmful effect of binge alcohol intake in the OLB group.

Third, tyrosine nitration frequently results in the misfolding of structural proteins and the inactivation of enzymes in nitrosative stress conditions [46,47]. The activity of catalase can be inhibited by tyrosine nitration [48]. There were low immune-positive signals in the liver tissue of the OLB group stained with anti-nitrotyrosine antibody (Figure 5e,f), which suggest the anti-nitrosative repertoire being activated by OLB might protect against free radical production caused by binge alcohol intake [5,34,49]. In the liver, catalase activity would be more important in the fasting condition [32]. We used 12 h fasted mice in this study, and the increased catalase activity might be, at least in part, responsible for low levels of AST and ALT. In chronic alcohol consumption, the free radical and catalase activity could be more important in brain tissue because catalase is the major enzyme to produce acetaldehyde from alcohol in the brain [48,50,51]. Thus, OLB can be useful to prepare a functional food to protect the liver, as well as brain, from the harmful effects of binge alcohol intake and chronic alcohol consumption.

## Figures and Tables

**Table 1:** List for genes and primers for RT-PCR analysis.

Gene Name	Abbreviation	Primer Sequence (5′ to 3′)
Alcohol dehydrogenase 1	ADH1	F; GCCGAAGCGATCTGCTAAT
		R; AGGTGCTGGTGCTGATAAAG
Cytochrome p450 enzyme 2E1	CYP2E1	F; CACAGCCAAGAACCCATGTA
		R; CATGAGAATCAGGAGCCCATATC
Aldehyde dehydrogenase 2	ALDH2	F; GTCTTCACAAAGGACCTGGATA
		R; GCCACTCCCTGACATCTTATAG
Superoxide dismutase 2	SOD2	F; CCACCGAGGAGAAGTACCACGAG
		R; CACACCGGAGACCAAATGATGTAC
Glutathione peroxidase 1	Gpx1	F; CCACCGAGGAGAAGTACCACGAG
		R; CTCCTTATTGAAGCCAAGCCAGCC
Cyclooxygenase 2	Cox2	F; TTTTCAGGCTTCACCCTAGATGA
		R; GAAGAATGTTATGTTTACTCCTACGAATATG
Tumor necrosis factor-α	TNF-α	F; CATATACCTGGGAGGAGTCT
		R; GAGCAATGACTCCAAAGTAG
Monocyte chemoattractant protein-1	MCP1	F; ACCTTTGAATGTGAAGTTGA
		R; CTACAGAAGTGCTTGAGGTG
Glyceraldehyde 3-phosphate dehydrogenase	GAPDH	F; GTGGCAAAGTGGAGATTGTTG
		R; TTGACTGTGCCGTTGAATTTG

**Table 2:** Concentrations of polyphenol compounds in freeze-dried water extract of oxidation-treated leaves (OL) and branches (BE) (ND, not detected).

	(+)-Catechin	Taxifolin	Quercetin	Chlorogenic Acid	Ferulic Acid
OL (µg/g)	56.2 ± 5.2	3.45 ± 0.5	54.7 ± 4.2	21.1 ± 4.2	44.5 ± 3.8
BE (µg/g)	268.9 ± 21.3	4.7 ± 0.8	ND	ND	35.7 ± 8.0

## Collectively

We established a method for using the leaves and branches from *H. dulcis* as functional substances with anti-hangover and liver protective properties during alcohol binge eating and chronic alcohol consumption. Moreover, our findings suggest that the long-term intake of OLB could protect the brain from oxidative or nitrosative stress because catalase activity is protected by OLB ingestion.
